# Relocating pediatric, hospital-based care towards preventive care: a qualitative study on modular care provision for children with developmental disabilities

**DOI:** 10.1007/s00431-026-06806-9

**Published:** 2026-02-23

**Authors:** Vincent J. T. Peters, Eva Caspers, Bert R. Meijboom, Sonja Hoofwijk, Inge N. E. Verbeek, Carin M. Delsman-van Gelder, Levinus A. Bok

**Affiliations:** 1 Department of Information Systems and Operations Management, Tilburg School of Economics and Management, Tilburg University, Tilburg, the Netherlands; 2 Department of Tranzo, Tilburg University, Tilburg, the Netherlands; 3 Department of Youth Healthcare, GGD Brabant-Zuidoost, Eindhoven, the Netherlands; 4 Department of Pediatrics, Máxima Medisch Centrum, Veldhoven, the Netherlands

**Keywords:** Relocation of care, Pediatric care, Youth public health care, Service modularity, Developmental disabilities

## Abstract

The aim of this study is to identify tasks and requirements for relocating healthcare for children with developmental disabilities in the Netherlands using a modular perspective. We conducted semi-structured interviews with three pediatricians and four youth public health physicians providing care for children with developmental disabilities in a southeastern region of the Netherlands. Additionally, we conducted ten practice observations. The data were analyzed using thematic analysis. We were able to visualize healthcare provision for children with developmental disabilities. This enabled us to identify modules—collections of tasks—which revealed opportunities and requirements to relocate certain care tasks from pediatricians to youth public health physicians. *Conclusion*: This study identified relocation opportunities for healthcare provision for children with developmental disabilities from pediatric care towards preventive care revealing duplication in care delivery. Also, this study identified five organizational requirements for relocation of care: adequate information and communications technology systems, clear agreements between parties involved, financial arrangements, knowledge and skills, and a shared desire. Relocation offers several potential benefits such as reducing workload and costs and improving care coherence and patient and family satisfaction.

**Table:** 

What is Known: • Relocating care—shifting tasks from pediatric care to primary or preventive care– is increasingly used to reduce costs, with evidence showing that such shifts can maintain care quality while lowering risks and expenses.
What is New: • We identify the organizational requirements for relocation of healthcare for children with developmental disabilities by revealing which tasks can be relocated and to whom.

**Supplementary Information:**

The online version contains supplementary material available at 10.1007/s00431-026-06806-9.

## Introduction

Health (system) policy measures have increasingly focused on shifting care from hospitals to primary and/or preventive care to contain rising healthcare costs [1, 2]. This is known as the relocation of care, but others use the term substitution of care [1]. This concept describes shifting tasks from one type of care setting or professional role to another, for the same patient population [3]. For example, shifting routine developmental and medical screening from costly hospital care to more affordable primary or preventive care. In the Netherlands, the healthcare system is organized across three levels of care: preventive, primary, and secondary care. Preventive care focuses on disease prevention and early identification of health risks (e.g., vaccination, screening, youth public health services performed) performed by youth public health professionals; primary care provides first-contact, accessible, and largely curative care performed by general practitioners and community nurses; and secondary care comprises specialist, hospital-based care performed by pediatricians, in the Dutch healthcare system typically accessed via referral. Primary and/or preventive care is oftentimes able to deliver the same quality and accessibility of care for certain patient groups, at lower costs and with fewer risks of adverse events [4, 5]. Evidence for the benefits of relocating hospital care to primary and preventive care is found, for example, in cardiology care [4], cancer care [6], dermatology care [7], and orthopedic care [8].

Healthcare professionals themselves often perceive relocation of care as complex because they are unaware of the skills and capabilities of professionals at different levels of care [9]. This lack of mutual understanding is reinforced by limited opportunities for real-time interaction, which constrain informal communication and trust-building across care levels. As a result, they tend to share less with professionals at other levels of care, which is also a result of insufficiently interactive and interconnected information systems [3]. This can lead to professionals performing additional tasks themselves instead of sharing and delegating responsibilities with/to primary and preventive care professionals [3, 9]. Identifying care tasks to be relocated and the organizational requirements for relocating care is a way to avoid such undesirable effects [7].

The modularity concept can be used to identify the tasks and requirements for realizing relocation of care. Conceptually, modularity can help healthcare professionals, patients, and families to recognize units of care in terms of the identification of individual parts of care (i.e., components and modules) that are provided, at what moment in time, and by whom [10]. Components comprise individual healthcare tasks, whereas modules resemble a collection of components with a specific function [11]. A modular service architecture visualizes the deconstruction of healthcare provision into modules and components [10], revealing opportunities to reduce duplication, identify unaddressed care needs, and improve effective use of available resources [10].

The aim of this study is to identify opportunities and requirements for relocation of healthcare for children with developmental disabilities (DD) in the Netherlands. We focused on DD because it represents a group of children with conditions that require long-term care and support, including routine developmental surveillance, protocolized medical screening, parent guidance, and coordination with schools and municipalities. Such tasks are well suited to preventive care, while curative services remain essential for complex diagnostics and medical treatment [12]. Therefore, we address the following research question: *How can a modular perspective help identify the tasks and organizational requirements for relocation of healthcare for children with developmental disabilities?*

## Methods

### Study design

The consolidated criteria for reporting qualitative research (COREQ) [13] were used as a guideline for this qualitative interview study. The Institutional Review Board of Tilburg University decided that ethics approval was not required because no patients were involved in this study. The research was conducted in accordance with the Declaration of Helsinki.

### Recruitment and sample

This study was conducted in close collaboration with a teaching hospital in the southeast of the Netherlands to collect data on healthcare provision for children with DD. We aimed to identify which healthcare tasks are delivered by pediatricians (secondary, hospital-based care) and which parts of (duplicate) care could be relocated to youth public health physicians (YPHP) (preventive care) in the care region where the hospital is active; approximately 500,000 people live in this region. In April and May 2023, we contacted pediatricians employed by the Dutch teaching hospital and YPHPs employed by the Municipal Health Service in the region under study. In the Netherlands, pediatricians and YPHPs have distinct, formally regulated roles within the Dutch healthcare system. Pediatricians provide specialized, curative healthcare for children in hospitals. YPHPs provide preventive care services such as growth monitoring, screening, vaccination, and health promotion. Together, they ensure that children with DD receive comprehensive support from early detection through long-term management, with coordinated care and timely referral to specialized care and support services whenever necessary. All pediatricians and YPHPs providing care for these children in the region under study expressed their interest in participating in our study. Seven interviews with pediatricians and YPHPs were conducted (Table 1); these seven individuals represent the complete set of professionals with relevant expertise in the region under study, making the sample exhaustive rather than selective.

**Table 1:** Respondent characteristics

Respondent	Gender	Age	Profession	Years of experience
Respondent A	Female	55–60	Youth public health physician	28–30
Respondent B	Female	40–45	Youth public health physician	13–15
Respondent C	Female	45–50	Youth public health physician	13–15
Respondent D	Female	45–50	Youth public health physician	18–20
Respondent E	Female	30–35	Pediatrician	0–2
Respondent F	Female	30–35	Pediatrician	3–5
Respondent G	Female	40–45	Pediatrician	5–8

### Data collection

#### Interviews

We conducted seven in-person semi-structured interviews in April and May 2023, each lasting approximately 60 min. We obtained verbal consent from all participants. An interview topic guide based on scientific literature [1, 4, 7, 9, 10] was used for the interviews (Appendix 1). The topic guide included topics like professionals’ routine consultations and opportunities and requirements for relocation of care. Interviews were audio-recorded and transcribed verbatim by one researcher (EC). Participants reviewed their own transcripts to check for accuracy and resonance with their experience, which improved the reliability of our interpretations [14].

#### Observations

One researcher (EC) conducted ten unstructured practice observations (five for each healthcare professional) during consultations to observe the actual work practices and working methods of both pediatricians and YPHPs. Each observation lasted half a day. This deepened our understanding of the daily practice of care provision. We obtained verbal consent from parents of children with DD and healthcare professionals prior to our observations. The observations focused on the question “*What elements of healthcare does the healthcare professional provide during the consultation?*” We used an observation template to write down our observation notes. This data was used to validate the information gathered from the interviews.

### Data analysis

The final data consisted of interview transcripts and observation notes which helped us to (1) acquire information on the professional’s perspective on care provision and potential for relocation of care, (2) obtain a better impression of the daily practice of care provision, and (3) gain insight into the actual practices of each professional. A coding reliability approach to thematic analysis was carried out [15] in which theory informs the early development of themes and codes. We followed this approach because it allowed us to identify opportunities for the relocation of care. We used theory on modularity and relocation of care in the early development of the codes and themes. Coding was carried out by two researchers (EC and VP). For example: we used the national guideline for children with DD [16] to assign distinct parts of the consultation from each professional as modules. We then identified the components of care currently provided by the professionals as healthcare tasks provision belonging to a certain module. Participants did not express themselves in modularity terms; instead, we used modularity as a perspective that guided interpretation of the data. This kind of modular interpretation of healthcare contexts has been applied frequently in the existing healthcare modularity literature [10, 11, 17, 18]. The combined interview and observation data allowed us to describe and interpret the practices of healthcare professionals in modular terms and identify tasks and organizational requirements for relocation of care.

## Results

To explore opportunities for relocation of care, the modular service architecture of pediatricians and YPHPs was created.

### Modular care provision for children with developmental disabilities

Both pediatricians and YPHPs mentioned that distinct parts of their respective consultation represent specific functions; these parts are based on the national guideline for children with DD as developed by the Dutch Pediatric Association [16]. We labeled these distinct parts of each professional’s consultation as modules (e.g., “posture,” “neurological and motor skills”). In turn, care tasks that belong to a specific module are interpreted as components (e.g., the component “examination of feet” is part of the module “posture” as offered by the YPHP; the component “reflexes” is part of the module “neurological and motor skills” as offered by the pediatrician). The healthcare professionals stated that consultations are personalized depending on, for example capabilities of the patient, the patient’s age, eating habits, potty training for toddlers, and sexual education and stimulant use for adolescence.

**Fig. 1:**
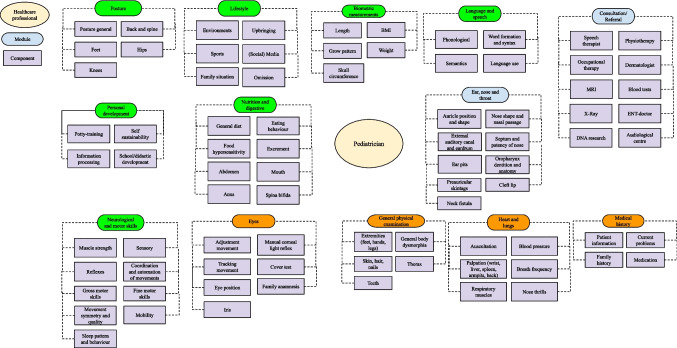
Modular service architecture pediatrician

**Fig. 2:**
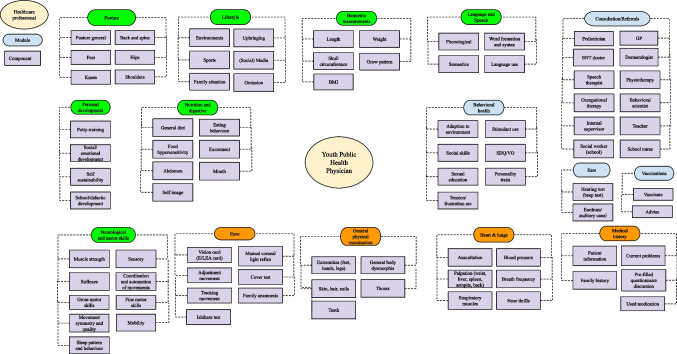
Modular service architecture youth public health physician

Using the identified modules and components of each healthcare professional, we constructed the modular service architecture of each healthcare professional (Figs. 1 and 2). These figures provide a visualization of all tasks offered by both pediatricians and YPHPs. Modules marked in green qualify for relocation (e.g., “biometric measurements,” “language and speech,” “posture” whereas modules marked in orange would qualify based on duplication but are intentionally retained because they serve a distinct function despite duplication (e.g., “medical history” to start the conversation). Some of these orange modules additionally require a supplementary check in the hospital setting despite relocation potential (e.g., “heart and lung assessment”). Modules marked in blue indicate modules that are unique to each healthcare professional.

### Organizational requirements for relocation

Using the modular service architectures, we were able to recognize what care tasks can be relocated. In what follows, we address five organizational requirements for the relocation of care: adequate information and communication and technology (ICT) systems, clear agreements between parties involved, financial arrangements, knowledge and skills, and a shared desire of professionals involved.

#### Presence of adequate ICT systems

Respondents unanimously agreed that adequate ICT systems, not necessarily an electronic health record, are important for realizing communication between pediatricians and YPHPs. Currently, many different information systems and communication methods exist in preventive, primary, and secondary care. One of the primary communication methods is digital information systems, which allow for referrals, requesting diagnostics, and consultations. While most referrals are created and transferred via the digital information system, professionals still have the possibility of communicating through more traditional communication channels such as email, physical (referral) letters, or phone calls. In some cases, healthcare professionals even preferred these methods, especially for urgent or complex cases. However, the preference for communication methods varies among professionals. Challenges for YPHPs in sharing patient information exist due to a lack of pre-existing parental consent forms and concerns about the security of the email system used by the regional public health services. Therefore, some professionals opted for sending patient information via traditional letters instead: “*I need to have a consent form that parents can sign. I created that form myself because the regional public health services do not provide one. And that must be sent by letter because the regional public health services do not have a secure mail environment.*” – YPHP 2.

#### Keeping agreements made between healthcare professionals involved

Relocation of care is only possible if healthcare professionals involved (get to) know each other, trust each other, speak each other’s language, and can hold each other accountable for keeping agreements made. There appears to be ambiguity and lack of clarity regarding who performs specific tasks in care provision for children with DD. None of the respondents mentioned documents or protocols that describe task division among pediatricians and YPHPs: *“I guess it is not really clear. I do offer some medical provision like biometric examination* [module]*, and the pediatrician is available as a back-up. … But I do feel more responsible for the paramedical part.*” – YPHP 1, and “*That is a tough question because I do not know what the YPHP actually does.*” – Pediatrician 3. Pediatricians also noted that it is often unclear who to contact and how. Finding and reaching YPHPs—especially for older children in different schools—can be difficult because pediatricians do not always know where YPHPs are located: “*Once you have contact with a colleague* [YPHP]*, the contact goes very well. However, the YPHPs are located in many different places, they are quite regularly hard to reach, and it is also not clear which YPHPs are working in which school.*” – Pediatrician 1. Pediatricians also argued that it is important to explain to all professionals, patients, and families involved, which syndromes and ailments are treated by whom to create clarity and transparency. For example, the YPHP assesses a child’s growth, regular vaccinations and psychosocial development. By law, YPHPs are not allowed to prescribe medication or order diagnostics like X-rays or blood examinations; they do refer to general practitioners or pediatricians. The pediatrician diagnoses and treats medical conditions such as pneumonia and chronic diarrhea.

#### Concerns about financial arrangements

All respondents emphasized that adequate financial arrangements are required to realize the relocation of care from pediatricians to YPHPs for children with DD. However, concerns about the availability of funding and personnel for adequate care provision were raised by all YPHPs. The YPHPs are funded by Dutch municipalities, while the pediatricians are funded by health insurance companies (based on diagnose-treatment activities for patients) and the national government. Some YPHPs state there is insufficient funding and staff to provide semi-annual, annual, or biennial follow-ups if care activities were to be relocated.

#### The presence of sufficient knowledge and related skills

Respondents mentioned that relocation of care is only possible when YPHPs have sufficient knowledge and relevant treatment skills for the complaints and conditions of children with DD. Overall, pediatricians spoke highly about the screening ability and examinations of the YPHP. They believed YPHPs have the knowledge and skills to identify potential health problems, and some pediatricians even consider YPHPs better suited for providing pedagogical assistance. They valued the role of YPHPs in signaling potential health issues during preventive screenings in preventive care: “*The follow-up examinations of the municipal health services are really good, in my opinion. I think they frequently examine by using questionnaires and physical examinations. I am very positive about that.*” – Pediatrician 2.

#### A shared desire to relocate care

Respondents argued that they must complement rather than duplicate each other, because otherwise the added value of pursuing relocation is diminished. While pediatricians believed they deliver patient-centered care, YPHPs expressed that current care delivery is tailored too much to the general pediatric population (i.e., children without disabilities). They question whether the current setup adequately caters to children with DD: “*Many parents are frustrated because they feel misunderstood, as the questionnaires are not suited to for children with DD, and sometimes they do not even complete them. Special education cannot be compared to regular education.*” – YPHP 2. YPHPs acknowledged that pediatricians have more resources and specialized knowledge to perform additional medical examinations and detect conditions, therefore being able to provide care that is better tailored to the specific patient population at hand. All respondents assumed that parents perceive YPHPs as generalists and pediatricians as medical specialists. YPHPs preferred to refer patients to pediatricians when cases become too complex or require specialized expertise. Relocation of care is only possible if professionals are willing to relocate care and accept responsibility for specific tasks, as such shifts affect professional roles and scope of practice, and must not compromise quality of care.

## Discussion

The findings show that healthcare provision for children with DD by pediatricians and YPHPs can be systematically deconstructed into a modular service architecture, as reported by others [10, 11]. These visualizations facilitate relocation of care since healthcare professionals could select which modules or components to include in follow-up examinations. This is in line with previous research [11] and led to insights into each professional’s work practices. Furthermore, the visualization of the work practices brings transparency to the pediatricians’ and YPHPs’ respective roles, responsibilities, and tasks, addressing current ambiguity and lack of clarity. For example, we discovered that some professionals have misconceptions, assuming that “only” biometrical measurements overlap. However, multiple duplicates were identified, indicating that these professionals can perform certain tasks interchangeably, suggesting relocation possibilities.

**Fig. 3:**
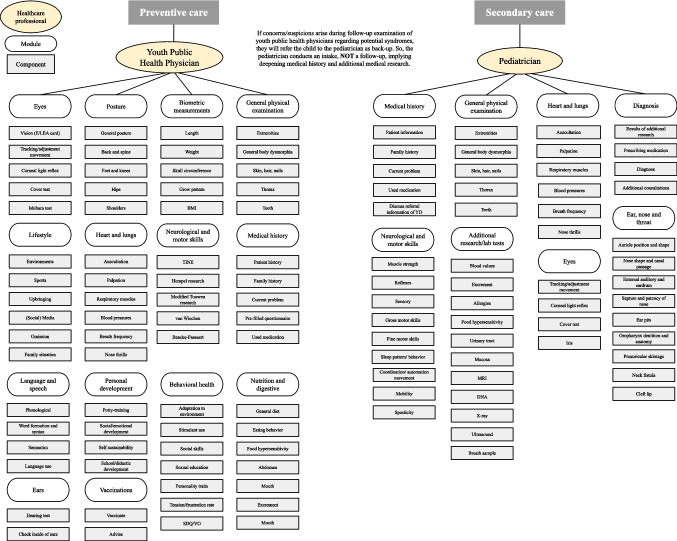
Future scenario for relocation of care

We show that the relocation of some parts of healthcare for children with DD is possible. Examinations [modules] such as “personal development” and “nutrition and digestive” qualify for relocation of care as they comprise general procedures and knowledge that are common among the healthcare professionals involved [3, 12]. Not all modules necessarily need to be relocated; some may be structured as age-dependent modules performed at specific ages by either a pediatrician or YPHP. Complete relocation of care for qualifying modules appeared not to be feasible because YPHPs are prohibited from performing specific care activities by professional rules and/or law, such as prescribing and administering medications. Building on those findings, Fig. 3 presents a future scenario in which relocation of care is considered based on the duplicate modules offered by pediatricians and YPHPs.

This study identified five organizational requirements for relocation of care: adequate ICT systems, clear agreements between parties involved, financial arrangements, knowledge and skills, and shared desire. First, there appeared to be inconsistency in information sharing, with referrals and updates often lacking consent, sufficient information, or timelines [9]. Interoperable ICT systems can play a pivotal role in this regard. Also, clear agreements between the professionals involved in secondary and preventive care are currently lacking, potentially leading to duplication of care, excessive medical consumption, or gaps in care. A distinct care pathway should be developed that explicitly includes all care professionals involved in care provision for children with DD, incorporating regular joint face-to-face meetings as an integral component to ensure effective alignment, avoid duplications, and adverse effects [3] as advocated by the Dutch Pediatric Association [19]. Moreover, inadequate funding poses challenges to pursuing relocation, in line with findings by previous research [6]. Regional public health services lack the necessary budget and staff to provide additional care services resulting from the relocation. Increasing the (medical) workforce in preventive care and their funding is therefore an important prerequisite for relocation to succeed.

### Limitations and future research

Our study has some limitations. First, the sample size of our study is relatively small and limits generalizability. However, these seven individuals resemble the set of healthcare professionals providing care for children with DD in the region under study; this has led to data saturation. Future research could explore comparisons across different countries and care systems to justify its relevance beyond the Dutch healthcare system. Second, children’s and families’ perspectives should be included to understand how they perceive the relocation of care, as their views are essential for realizing a complete and nuanced picture of its potential and impact [20].

## Conclusion

This study shows that a modular perspective helps identify where elements of healthcare for children with DD can be relocated from hospitals to primary and/or preventive care. By revealing duplicate tasks like “biometric measurements” in current care delivery, it highlights concrete opportunities for relocation that can reduce professionals’ workload, improve the coherence of care for patients and consequently patient and family satisfaction, and reduce overall healthcare costs.

## Supplementary Information

Below is the link to the electronic supplementary material.

ESM 1 Supplementary Material 1 (DOCX 19.6 KB)

ESM 2 Supplementary Material 2 (DOCX 20.3 KB)

## Data Availability

The data that support the findings of this study are available from Tilburg University, but restrictions apply to the availability of these data. The data are, however, available from the authors upon reasonable request and with the permission of the Ethics Review Board of Tilburg University.
