# Supplementary material for: Relocating pediatric, hospital-based care towards preventive care: a qualitative study on modular care provision for children with developmental disabilities
Source: Eur J Pediatr. 2026 Feb 23;185(3):148. doi: 10.1007/s00431-026-06806-9 (PMC12929245; doi:10.1007/s00431-026-06806-9)
Supplement: Supplementary file 1 — Supplementary Material 1 (DOCX 19.6 KB) [file 431_2026_6806_MOESM1_ESM.docx]

**Appendix 1. A 32-item checklist for reporting qualitative studies (COREQ)**

| **Item** | **Description** | **Check** |
| --- | --- | --- |
| **Domain 1: Research team and reflexivity** | | |
| *Personal characteristics* | | |
| 1. Interviewer | Which author/s conducted the interviews and/or observations? | Author 2 |
| 2. Credentials | What were the researcher’s credentials? | Author 1: Dr.  Author 2: MSc  Author 3: Prof. dr. ir.  Author 4: MSc  Author 5: MSc  Author 6: Dr.  Author 7: Dr. |
| 3. Occupation | What was the occupation at the time of the study? | Author 1: Assistant professor  Author 2: Master’s student  Author 3: Endowed professor  Author 4: Youth public health physician  Author 5: Pediatrician – Social pediatrics  Author 6: Pediatrician – Pediatric neurologist  Author 7: Pediatrician |
| 4. Gender | Was the researcher male or female? | Author 1: Male  Author 2: Female  Author 3: Male  Author 4: Female  Author 5: Female  Author 6: Female  Author 7: Male |
| 5. Experience and training | What experience or training did the researcher have? | Courses on Qualitative Research Methods, experience with peer reviewed scientific output on healthcare, modularity and clinical studies. |
| *Relationship with participants* | | |
| 6. Relationship established | Was a relationship established prior to study commencement? | A professional relationship was established with the pediatricians, based on previous collaborations, ahead of the research. |
| 7. Participant knowledge of the researcher | What did the participants know about the researcher? | The personal interest of the researchers and purpose of the study was explained before the data collection started. |
| 8. Researcher characteristics | What characteristics were reported about the researcher? | Interest in research topic, occupation, and reason for research. |
| **Domain 2: Study design** | | |
| *Theoretical framework* | | |
| 9. Methodological orientation and Theory | What methodological orientation was stated to underpin the study? | Coding reliability approach to thematic analysis |
| *Participant selection* | | |
| 10. Sampling | How were participants selected? | Purposive sampling |
| 11. Method of approach | How were participants approached? | Telephone, face-to-face, e-mail |
| 12. Sample size | How many participants were in the study? | 7 |
| 13. Non-participation | How many people refused to participate or dropped out? Reasons? | 0 |
| *Setting* | | |
| 14. Setting of data collection | Where was the data collected? | Hospital (in-person) and youth public healthcare organization digital (via Zoom) |
| 15. Presence of non-participants | Was anyone else present besides the participants and researchers? | No |
| 16. Description of sample | What are the important characteristics of the sample? | Gender, age, profession, years of experience |
| *Data collection* | | |
| 17. Interview guide | Were questions, prompts, guides provided by the authors?  Was it pilot tested? | The interview topic guide is added to the manuscript; the topic guide was pilot tested and discussed within the study team. |
| 18. Repeat interviews | Were repeat interviews carried out? If yes, how many? | No repeat interviews were carried out. |
| 19. Audio recording | Did the research use audio recording to collect the data? | Yes |
| 20. Field notes | Were field notes made during and/or after the interview or observation? | Yes |
| 21. Duration | What was the duration of the interviews or observation? | Interviews: 60 minutes |
| 22. Data saturation | Was data saturation discussed? | Data saturation was discussed within the research team. |
| 23. Transcripts returned | Were transcripts returned to participants for comment and/or correction? | Each transcript was returned to the individual corresponding participant. We received no comments and corrections. |
| **Domain 3: Analysis and findings** | | |
| *Data analysis* | | |
| 24. Number of data coders | How many data coders coded the data? | Two (Author 1 and Author 2) |
| 25. Description of the coding list | Did authors provide a description of the coding list? | The coding list is described in Data analysis part of the Methods section. |
| 26. Derivation of themes | Were themes identified in advance or derived from the data? | Themes were identified in advance, but we also made use of a code called ‘other’ in which relevant other themes were categorized. This is in line with the coding reliability approach to thematic analysis. |
| 27. Software | What software, if applicable, was used to manage the data? | Microsoft Word and Microsoft Excel |
| 28. Participant checking | Did participants provide feedback on the findings? | We asked the participants to reflect on the findings of the study and received no comments or corrections. The participants mentioned that they recognized the modular way of working and potential for substitution. |
| *Reporting* | | |
| 29. Quotations presented | Were participant quotations presented to illustrate the themes/findings? Was each quotation identified? | We used various quotations from our participants. Moreover, we created two figures to illustrate our results. |
| 30. Data and findings consistent | Was there consistency between the data presented and the findings? | We present an analytic story where we highlight the key themes of the study; modular decomposition and organizational requirements for substitution. |
| 31. Clarity of major themes | Were major themes clearly presented in the findings? | We present two major themes in the Results section. |
| 32. Clarity of minor themes | Is there a description of diverse cases or discussion of minor themes? | The description of the two healthcare professionals involved are presented in the Results section of the study. Minor themes are presented in the running text as part of each major theme. |
