# Supplementary material for: Relocating pediatric, hospital-based care towards preventive care: a qualitative study on modular care provision for children with developmental disabilities
Source: Eur J Pediatr. 2026 Feb 23;185(3):148. doi: 10.1007/s00431-026-06806-9 (PMC12929245; doi:10.1007/s00431-026-06806-9)
Supplement: Supplementary file 2 — Supplementary Material 2 (DOCX 20.3 KB) [file 431_2026_6806_MOESM2_ESM.docx]

**Appendix 2. Interview topic guide**

**1. Demographics**

Could you briefly introduce yourself and go over the following topics:

- Your age
- Your gender
- Your position and role within healthcare provision for children with developmental disabilities
- Your experience in healthcare provision for children with developmental disabilities

**2. Organization of Care**

2.1 Based on the regional public health services organization’s website, health assessments are conducted for children with developmental disabilities aged 5-6 years and 10-11 years. The assessment for 5-6 year olds lasts 45 minutes, while the one for 10-11 year olds lasts 30 minutes.

- Why are these assessments conducted at these age categories?
- Why is there a difference in the duration of these assessments?

2.2 What components (tests) are included in the health assessment?
2.3 What is the purpose of each test within the health assessment?
2.4 Could you briefly describe the procedures involved in each test? What materials are used for these tests?
2.5 Do the tests or procedures differ based on age?
2.6 Are there specific protocols or guidelines available on how to conduct certain tests or procedures?

2.7 Do you agree with how the health assessments are currently organized?

- If so/not, why?

2.8 Do you agree with who performs the health assessments and where they take place?

- If so/not, why?

**3. Coordination of Care**

3.1 Before the health assessment takes place, parents or guardians complete two questionnaires. Is the information from these questionnaires used solely by your team, or is it also shared with other healthcare professionals (e.g., pediatricians in hospitals)?
3.2 What kind of information exchange related to the health assessment occurs with pediatricians in hospitals? Could you provide some examples?
3.3 How does this information exchange take place?
3.4 Which information exchange(s) is/are most important?

3.5 Do patient or progress meetings take place in which the child’s care plan is discussed?
3.6 Do these meetings take place within your team?

- And also outside your team? (e.g., with pediatricians from hospitals)

3.7 How do you experience the communication and information exchange with hospital pediatricians?

- Specifically, is communication easily established, and is the information exchanged complete, accurate, and up-to-date? (e.g., language barriers, differences in medical jargon)

3.8 How are work processes and activities coordinated between involved healthcare professionals (pediatrician and youth healthcare physician)?

3.9 Who is designated as the coordinator responsible for the care of children with developmental disabilities?

- How is this coordination managed?
- Who schedules the health assessments?
- And who checks whether they have taken place?

3.10 What is your opinion of the current coordination of care for children with developmental disabilities?

- And why?

3.11 Do you see opportunities for improvement regarding the coordination of tasks between involved healthcare professionals (pediatrician and youth healthcare physician)?

- Why?

3.12 As a youth healthcare physician, you have the authority to refer children to various forms of youth care, including pediatric care. How do you experience the referral process?

3.13 To your knowledge, are there specific rules that may affect collaboration between hospitals and the regional public health services organization?

**4. Substitution of Care**

4.1 Are there, in your opinion, clear agreements/protocols regarding which healthcare professional provides which part of care?

- If so/not, why?

4.2 If certain responsibilities were to shift between healthcare professionals, this would mean that the role as a 'gatekeeper' becomes more significant. It may also result in more frequent contact with involved healthcare professionals than before.

- Do you think there is enough trust between the involved healthcare professionals (pediatrician and youth healthcare physicians) to discuss potential differences of opinion?

4.3 If tasks were to shift between healthcare professionals, this might also mean that you would need to perform additional tests and procedures during health assessments.

- How would you feel about this in terms of workload, time, and scheduling?
- Do you feel that you have all the necessary materials available to perform these assessments and create the most accurate representation of the child?
- Why or why not?

4.4 What is your opinion on shifting follow-up assessments from pediatricians to youth healthcare physicians?
4.5 The training of youth healthcare physicians and pediatricians differs in terms of specialization, which may result in differences in knowledge and expertise.

- Do you believe that both healthcare professionals can arrive at equivalent diagnoses during these health assessments?

**5. Wrap-up**

Is there anything else you want to share with us regarding the topic of the interview at hand that was not discussed during the interview?
